# Homœopathy in Venereal Diseases

**Published:** 1888-09

**Authors:** 


### BOOK NOTICES.

Homœopathy in Venereal Diseases. By Stephen Yeldham, L. R. C. P., Ed., M. R. C. S., Eng. Edited by Henry Wheeler, L. R. C. P., Lond., M. R. C. S., Eng. Fourth edition. London: E. Gould & Son; New York: Bœricke & Tafel. 1888.

It is difficult for a homœopathist to understand the reason for the publication of such books as this monograph of Dr. Yeldham's. Its treatment of the pathology and clinical history of venereal diseases is brief and unsatisfactory, its discussion of therapeutic measures is even worse, being neither homœopathic nor allopathic. It is, in fact, a mixture which is useless to all. The book is neatly printed.

